# Utilizing Excess Resin in Prepregs to Achieve Good Performance in Joining Hybrid Materials

**DOI:** 10.3390/polym17121689

**Published:** 2025-06-18

**Authors:** Nawres J. Al-Ramahi, Safaa M. Hassoni, Janis Varna, Roberts Joffe

**Affiliations:** 1 Department of Engineering Sciences and Mathematics, Luleå University of Technology, 97187 Luleå, Sweden; janis.varna@associated.ltu.se (J.V.); roberts.joffe@ltu.se (R.J.); 2 Institute of Technology-Baghdad, Middle Technical University, Baghdad 10066, Iraq; dr.safaa1970@mtu.edu.iq; 3 Institute of High-Performance Materials and Structures, Riga Technical University, LV-1048 Riga, Latvia

**Keywords:** double cantilever beam, Al–Si-coated boron steel, metal–composite adhesive joints, epoxy adhesives, adhesive layer thickness

## Abstract

This study investigates the fracture toughness of adhesive joints between carbon fiber-reinforced polymer composites (CFRP) and boron-alloyed high-strength steel under Mode I and II loading, based on linear elastic fracture mechanics (LEFM). Two adhesive types were examined: the excess resin from the prepreg composite, forming a thin layer, and a toughened structural epoxy (Sika Power-533), designed for the automotive industry, forming a thick layer. Modified double cantilever beam (DCB) and end-notched flexure (ENF) specimens were used for testing. The results show that using Sika Power-533 increases the critical energy release rate by up to 30 times compared to the prepreg resin, highlighting the impact of adhesive layer thickness. Joints with the thick Sika adhesive performed similarly regardless of whether uncoated or Al–Si-coated steel was used, indicating the composite/Sika interface as the failure point. In contrast, the thin resin adhesive layer exhibited poor bonding with uncoated steel, which detached during sample preparation. This suggests that, for thin layers, the resin/steel interface is the weakest link. These findings underline the importance of adhesive selection and layer thickness for optimizing joint performance in composite–metal hybrid structures.

## 1. Introduction

The use of high-strength structural adhesives to bond metal with composite materials has become a key solution in the ongoing optimization of the automotive industry in terms of vehicle lightness and safety. The drive to develop lighter structures in transportation vehicles stems from multiple motivating factors, encompassing both environmental and economic considerations. These factors are intrinsically linked to the goal of reducing fuel consumption. These developments are particularly crucial for industries involved in transportation, most notably aerospace, aeronautics, and automotive sectors. This weight-saving advantage is exemplified in the construction of cutting-edge commercial aircrafts, like the Airbus A380 and Boeing B787, wherein composite materials constitute approximately 25–50 wt % of the structural components [1]. In the realm of manufacturing intricate structures using different materials, the process of joining multiple parts becomes essential. Mechanical joints are commonly used within metal structures, whereas adhesive joining is the preferred method for polymer composites. Consequently, when incorporating composite materials into a structure, the conventional practice of employing mechanical joints must be replaced by adhesive joints [2]. In addition to their reduced weight [3], adhesive joints offer several advantages compared to mechanical joints. Firstly, mechanical joints are unsuitable for adherends thinner than 8 mm [4]. Moreover, the presence of holes for bolts and rivets in mechanical joints increases the concentration of stress, thereby weakening the composite structure. In contrast, adhesive joining ensures a more even distribution of stress throughout the joint [3]. Hence, it is imperative to possess the ability to exclusively employ adhesive bonding for the assembly of the principal substantial components of the aircraft [5]. To enhance the robustness of the connection, a better understanding of the mechanisms governing the initiation and progression of failure within the adhesive layer is necessary. Delamination stands as a prevalent failure mode that frequently affects structural components composed of laminated composites. This phenomenon exerts considerable influence on the mechanical properties of the composite parts, particularly by reducing their stiffness and strength. Delamination can be attributed to various factors, including manufacturing imperfections, fatigue, and stress concentrations arising from material discontinuity. These conditions generate interlaminar stresses, which constitute the stresses acting at the interface between laminas. If these stresses reach a certain threshold, delamination failure may ensue. To address this issue, one potential solution lies in the application of fracture mechanics. This involves determining the fracture toughness or critical energy release rate of the material [6,7]. Such methodology enables the separation of three separate failure modes. It is important to note that each of these crack extension modes corresponds to a specific crack tip stress intensity factor (K_I_, K_II_, K_III_) or strain energy release rate (G_I_, G_II_, G_III_). The material is assumed to have a linear elasticity. Consequently, the values of G can be deduced by evaluating the elastic work associated with the gradual closure of the crack [8,9].

Numerous research studies have addressed the various failure modes, namely Mode I, Mode II, and mixed mode, concerning the propagation of damage [9,10,11,12,13,14,15,16,17,18,19,20,21]. Crack expansion in a sizable plate subjected to plane loading was the subject of a theoretical and experimental examination conducted in [9]. The findings illustrated that under pure Mode II conditions, the crack extends at an angle of 70° that is approximately perpendicular to the orientation of the highest tangential load. In [10], an academic study proposed a simulation model for investigating the damage initiation and propagation in adhesive bonded joints under mixed-mode conditions (Mode I+ Mode II). The detection of damage initiation in the joints relied on a stress criterion, while the gradual degradation of joint stiffness was assessed using a fracture mechanics criterion for both the DCB and ENF specimens. In the study conducted in [11], an advanced virtual crack closure technique was employed to forecast the propagation of cracks in both single and multi-level delaminations, as well as debonding occurrences in composite adhesively bonded joints. The investigation encompassed DCB and ENF specimens. The investigation presented in [12] involves both experimental and numerical methods to analyze the DCB and ENF tests under loading conditions of pure Mode I, pure Mode II, and mixed Mode I/II. Additionally, the research delves into the study of debonding phenomena occurring in composite adhesively bonded joints under mixed-mode loading. To facilitate this analysis, the researchers employed a criterion that enables the determination of the critical energy release rate based on the mixing mode ratio. Consequently, this criterion allows for the calculation of the critical energy release rate for any given mixing ratio. In [13], the authors conducted mixed-mode fracture testing of DCB specimens of composite laminates and adhesive joints under jig load. The experimental results demonstrated that the G_IC_ values were roughly one-third of the G_IIC_ values. Additionally, it was observed that the G_IC_ values remained unaffected by the adherend thickness. Furthermore, the fracture resistance was found to be independent of the crack length. A comprehensive investigation was conducted on the interface of a double cantilever beam, utilizing cohesive crack modeling (CCM) as shown in [14]. This study aimed to predict the structural response under Mode I loading conditions. Various methodologies, including simple beam theory (SBT), enhanced beam theory (EBT), and finite fracture mechanics (FFM), were applied to compare their results and determine the most accurate predictor for the maximum load required for crack propagation. The findings revealed that SBT and EBT yielded less reliable estimations for the maximum load, whereas FFM and CCM demonstrated precise predictions. Additionally, the study confirmed that only the CCM and FFM approaches can effectively capture the transition between strength-governed and fracture energy-ruled debonding processes. A comprehensive investigation was conducted to enhance the beam model for DCB made of composite material. The study compared the results of one-dimensional analysis with two- and three-dimensional analyses, as presented in [15]. In order to analyze the cracked part, a shear-corrected classical beam theory was employed. On the other hand, the deformation of an uncracked part of the beam was analyzed using the concept of a beam on an elastic foundation, and the Saint Venant effects were taken into consideration. The findings of the study indicated that the strain energy release rates (SERR) for the intermediate state (neither plane strain nor plane stress) were underestimated when the plate solutions assumed a plane strain state. Conversely, they were overestimated when the beam and plate solutions assumed a plane stress state. Furthermore, when examining the SERR for specimen geometries within the intermediate state, there was no significant difference in the results for orthotropic specimens compared to isotropic specimens. However, a substantial disparity in the results was observed for unidirectional specimens oriented at ±45°. In a separate study described in [16], the Mode I fracture behavior and strength of adhesive bonded DCB specimens were investigated experimentally and numerically. The experimental results revealed that the crack transitioned from the lower adherend to the upper adherend within the adhesive layer at a kinking angle of 60°. Different energy methods used to calculate the SERR yielded similar values. In [17], an analytical and numerical model was developed to assess whether inserting weak stiffness layer could enhance the fracture resistance of a structure by changing the crack mechanism. The models were based on the J-integral method and assumed a linear dependency between steady-state fracture resistance and the number of cracks. The results showed significant improvement in fracture resistance when weak layers were added. An experimental and numerical study conducted in [18] investigated dissimilar adherend (composite-steel) DCB joints under pure Mode I. The study aimed to achieve pure Mode I behavior by matching the longitudinal strain distributions of both adherends at the bondline. The results demonstrated that using strain-based design criterion and curvature-based criterion in bi-material joints allowed for the attainment of pure Mode I. Additionally, when using the longitudinal strain-based criterion, the ratio between G_II_/G_I_ was reduced by a factor of 5 compared to the flexural stiffness-based criterion. An experimental and numerical investigation was presented in [19]. This study focused on the mechanical behavior and damage mechanisms of carbon fiber-reinforced thermoplastic composite (T700G/LM-PAEK). Mechanical properties were characterized using tensile, compressive, DCB, and ENF tests, revealing that T700G/LM-PAEK exhibits comparable or superior stiffness, strength, and interlaminar fracture toughness to similar thermoset composites. A 3D XFEM approach including an elastoplastic constitutive law accurately predicted the stress–strain responses and failure modes in open-hole tension and compression specimens, aligning well with experimental results and highlighting the importance of considering plasticity in modeling such materials. In [20], the study examined how temperature and loading rate affect Mode I and II interlaminar fracture toughness in CNT-enhanced CFRP. Samples with varying CNT areal densities were tested under different conditions, and fracture surfaces analyzed via SEM. Toughness increased with CNT content up to 1 g/m², then started to decrease at higher amounts of CNT. Fracture toughness rose linearly with loading rate for both modes. Temperature effects differed: Mode I toughness increased with temperature, while Mode II peaked at room temperature and dropped at higher levels. Another study [21] also investigated the effect of adhesive thickness on joint performance. This study showed that the fracture energy increased with an increase in the adhesive thickness; the fracture energy increased by two times when the thickness increased by three times.

In conclusion, the optimization of adhesive bonding techniques in the automotive industry represents a critical frontier in the ongoing activity of lightweight, efficient, and safe vehicles. The integration of high-strength structural adhesives to bond metal with composite materials offers a promising solution to the challenges posed by traditional mechanical joining methods. As research in this field advances, it is essential to continue exploring the complexities of adhesive joint behavior and delamination mechanisms to ensure the reliability and performance of future vehicle designs. The primary aim of the presented research is to enhance comprehension of crack propagation within the adhesive layer for dissimilar adherends. To achieve this objective, an experimental investigation approach was undertaken. To conduct the study, two different types of samples were utilized: one with a thin adhesive layer created by using the resin in the prepreg as an adhesive, and the other with a thick adhesive layer utilizing Sika Power-533 MBX (Sika Technology AG, Frankfurt, Germany) [22] as an adhesive. The impact of various parameters on the behavior of crack propagation was examined.

The presented research is motivated by the practical need to optimize the composite-steel joint manufacturing ensuring its sufficient durability and resistance to failure. An essential problem in joining separately manufactured composite and steel parts of complex geometry is a typical shape distortion in the composite part due to complex thermal–chemical–viscoelastic effects and other phenomena during curing and post-curing. A rather thick adhesive layer has to be used between them to ensure good shape fitting of both parts. The problem with the “incompatibility” of shapes can be partially eliminated by joining different parts simultaneously with the processing of the composite (so-called co-curing). In such cases, the composite part is manufactured by placing the prepreg on the steel part during curing and utilizing the excess of resin in the prepreg. The excess resin forms a thin adhesive layer and bonds metal to composite material. The compatibility of the resin (adhesive) and the composite is automatically satisfied whereas the compatibility with the steel is a problem to solve by material scientists.

This study presents a novel comparative investigation into the fracture toughness behavior (Mode I and Mode II) of composite-to-steel joints fabricated using two fundamentally different bonding strategies: co-curing with excess resin from prepreg and bonding with a commercial adhesive (SikaPower-533 MBX). Unlike previous work, which has often focused on either co-cured or secondarily bonded joints in isolation, this research directly contrasts the mechanical performance of joints with thin, in situ formed adhesive layers versus thick, industrial-grade adhesive layers, underlining the influence of adhesive thickness, material compatibility, and Al–Si coating presence. Two rather extreme cases are considered and evaluated. In the case when excess resin from prepreg is utilized, the adhesive layer is thin, and it has an excellent compatibility with the composite part and an unknown compatibility with the steel. In the second case, when Sika Power-533 MBX is used to bond two materials, a rather thick and very tough adhesive layer is created. This adhesive is compatible with the steel by design, whereas its compatibility with the composite is unknown. The influence of the adhesive thickness and the presence of Al–Si coating on the boron steel is addressed and potential improvements are suggested.

Conclusions from mechanical tests are confirmed by fractography and information available in the literature.

## 2. Materials and Sample Manufacturing

### 2.1. Materials

This study utilized a hybrid joint (composite–metal), including a composite (C) material (Prepreg T700/E445 with a 38% resin content (ET445 FAST resin system)) and a steel plate. The resin (matrix) in the prepreg was formulated for the fast hot-press technique, ensuring rapid and complete curing. In the case of the ET445 epoxy, it needs 7 min to reach full cure, provided that the temperature is maintained at or above 140 °C. For the metal adherend, two different steel grades were employed: Al–Si-coated boron steel “USIBOR 1500” (cS) and uncoated boron steel 22MnB5 (uS). To study the thick adhesive layer a commercial epoxy resin (Sika Power-533 MBX) was used. According to the datasheet, this particular adhesive is specifically designed for use in structural joints involving metals and thermoset based composite, such as CFRP, its properties have been optimized to enable oil absorption while maintaining its adhesive characteristics intact. The datasheet specifies that the adhesive possesses “impact-modified” properties, thus the application of certain mechanisms to increase its toughness. The mechanical properties of the composite materials, boron steel, and Sika Power-533 MBX are shown in Table 1.

**Figure 1:**
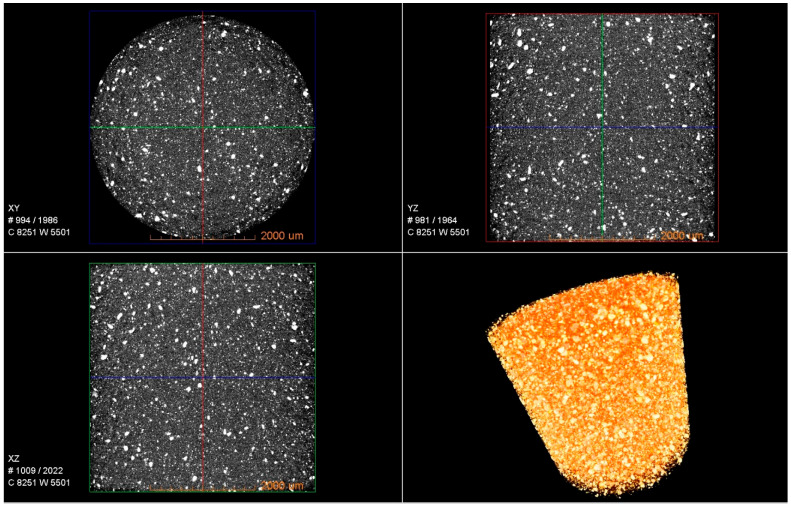
XMT image (original and segmented) of bulk adhesive sample showing uniformly distributed small particles within the adhesive as well as that there are no air bubbles.

In order to examine the adhesive material content as well as the air pockets in a large Sika adhesive sample, an analysis was carried out using the Zeiss Xradia 510 Versa X-ray microtomography (XMT) equipment. Figure 1 shows a three-dimensional image of the sample (4.5 × 4.5 × 4.5 mm^3^) in various representations. The images are segmented (based on density differences) in order to eliminate polymer, and it reveals the presence of uniformly distributed small particles within the adhesive. The nature of these particles remains unknown as it is not specified in the datasheet, but it may be concluded that the density of these particles is higher than that of the polymer. Moreover, there are no visible air pockets (which would be represented by black inclusions) within the sample, which is a good indicator of the robustness of the manufacturing process.

### 2.2. Sample Manufacturing

#### 2.2.1. Joining Methods

Two types of specimens were manufactured in this study, for DCB and ENF testing. As pointed out in [23], the equivalency of beam deflection in both the composite plate (CFRP) and boron-alloyed steel is crucial for achieving pure Mode I and Mode II in the adhesive. This is automatically guaranteed for symmetric DCB specimen (the same material and thickness of both beams); however, multi-material specimen has to be specially designed.

In order to achieve a symmetrical deflection of the beams in metal–composite DCB specimens, it is imperative to have an equal bending stiffness for the composite and metal beams. As steel stiffness and thickness are defined and cannot be changed, this requires determining the appropriate thickness for the composite laminate to be manufactured, so as to match the bending stiffness of the steel (D_composite_ = D_steel_). This leads to the equation (Equation (1)), which has to be satisfied.

(1) $$D_{steel}=D_{composite}\;\to \;\frac{E_{S}h_{S}^{3}}{12}=\frac{E_{C}h_{C}^{3}}{12}\;\to \;\frac{h_{S}}{h_{C}}=\sqrt[{3}]{\frac{E_{C}}{E_{S}}}$$

where h_S_ is the metal beam thickness, h_C_ is the composite laminate thickness, E_S_ is the steel modulus, and E_C_ is the composite modulus.

In compliance with the ASTM D5528-13 standard [24] for Mode II failure, it is required that the adherend of the CFRP composite laminate exhibit unidirectional characteristics, with delamination growth specifically occurring along the 0° direction. Given the modulus for both adherends as well as the thickness of the metal plate, it becomes possible to determine the thickness of the CFRP composite laminate by using Equation (1). The resulting thickness of the composite laminate is 1.463 mm, as the thickness of one prepreg layer is 0.3225 mm. It means that the composite beam should have 4.53 layers of prepreg with 0° orientation. Thus, a laminate consisting of five 0° layers was produced. To manufacture the composite plate, a pressure of 3 bars and a temperature of 160 °C for precisely 7 min were applied to consolidate the laminate and guarantee proper curing of the matrix. Within our specific manufacturing procedure, an application of pressure leads to a slight reduction in the thickness of the CFRP composite adherend. Consequently, the final thickness achieved was in close proximity to the calculated value, resulting in the attainment of almost pure Modes I and II.

There are several international standards for DCB Mode I testing, including the ASTM standard (ASTM D5528-01 2002), Japanese standard (JIS K 7086 1993), European standard (ASD-STAN preEN 6033 1995), and ISO standard (ISO 15024 2002) [25]. In this study, the standard (D5528-13) was used [24], while the standard (D7905M-14) [26] was used for the ENF (Mode II) test.

The metal and composite plates were joined using two methods. The first method utilized the resin (ET445 FAST) in the prepreg as an adhesive during the co-curing process, resulting in a thin adhesive layer (ET445 FAST with thickness < 0.2 mm). The second process uses the commercial epoxy adhesive (Sika Power-533 MBX) to join the already cured composite laminate with the steel, leading to the formation of a thick adhesive layer (with a thickness ~1.5 mm).

#### 2.2.2. Manufacturing of Specimens

**Figure 2:**
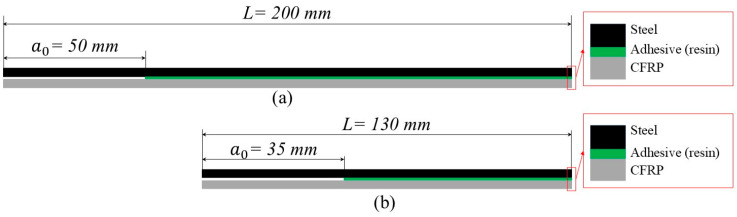
The (**a**) DCB and (**b**) ENF samples using the resin in the prepreg as an adhesive.

Two different methods were employed to produce specimens with the excess resin in the prepreg as the adhesive of the geometry shown in Figure 2.

**Figure 3:**
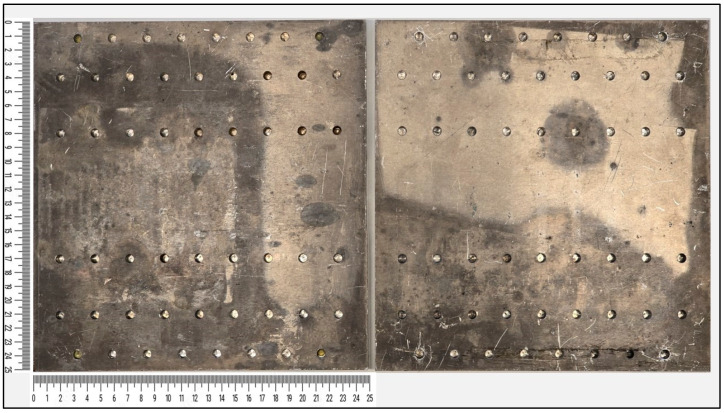
The mold that was used to manufacture the DCB and ENF samples.

In the first method for manufacturing the steel-composite and composite-composite DCB plates, a metal plate and an uncured composite plate, each measuring 200 mm × 200 mm, were joined together. The assembly was subjected to vacuum, pressure, and temperature, following the prepreg curing cycle. Then, a diamond wheel was utilized to precisely cut the joint plate, obtaining samples with dimensions of 20 mm × 200 mm for DCB test and for ENF test. The bondline thickness in both cases (thick and thin adhesives) was controlled using spacers positioned between the upper and lower parts of the mold, as illustrated in Figure 3. Thickness quantification was performed using optical microscopy on cross-sectioned samples to verify compliance with the design tolerances (e.g., 0.2 ± 0.05 mm).

A poor joint formed in the case when one of the adherends was uncoated steel, causing the metal plate to separate from the composite during the cutting process. This was not an issue for the coated steel–composite or composite–composite assembly as a stronger joint was achieved.

In the second method for manufacturing samples, the steel was first cut to the desired sample width and placed above the uncured composite prepreg (measuring 200 mm × 200 mm). The assembly was then joined by subjecting it to vacuum, pressure, and temperature (according to the prepreg curing cycle). But when joining steel and composite plates with Sika adhesive, the composite plate was manufactured first and then both the metal and the composite plates were first cut according to the desired sample width and subsequently placed in a mold (see Figure 3). Spacers were used to ensure a 5 mm gap between various metal–composite joints. The problem with damage introduced during cutting was eliminated this way.

Since the DCB tests (results are not presented here to keep the discussion brief) on specimens produced by cutting the coated steel–composite plate gave at least 10 to 20% lower fracture toughness G_IC_, only the manufacturing method that did not include cutting is used in following discussion for both types of adhesives.

**Figure 4:**
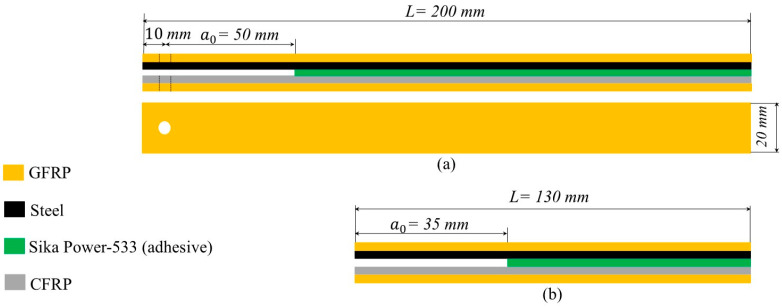
The (**a**) DCB and (**b**) ENF samples using Sika Power-533 MBX as an adhesive.

Using the Sika Power-533 MBX as adhesive, various adjustments were implemented in the design to account for the adhesive’s strength and the diverse properties of the substrates. The first modification was related to the excessive bending that is observed during the test. Thus, to avoid this issue, additional glass fiber laminate (GFRP) was attached on both surfaces of the DCB specimens to enhance the bending stiffness of beams. The second modification pertained to the slot for hinges. Typically, an open slot is created at the beginning of the sample to attach the hinge and establish the loading point. However, in this case, it led to premature failure by causing the specimens to slide out of the hinges during the test. To reduce the risk of the hinges sliding, it was decided to drill holes into the specimens and attach the hinges using screws. The final shape and dimension of the specimens are shown in Figure 4.

The DCB specimen utilized in this study was a standardized rectangular sample with constant thickness. A pre-existing crack was introduced at one end by inserting a thin (0.02 mm) non-adhesive film between the two substrates (at the middle of the adhesive layer) during the manufacturing process with a length equal to 50 mm (pre-crack length). The ENF specimen employed in this study was a rectangular sample with constant thickness, according to the ASTM standard [26] and the test method in [27]. The initial crack $a_{0\;}\geq 0.7\;L$ (where L is the span length which is equal to 50 mm), thus the initial crack length was 35 mm (the dimensions of the specimen were as follows: width = 20 mm, length = 130 mm).

## 3. Experimental Procedure

**Figure 5:**
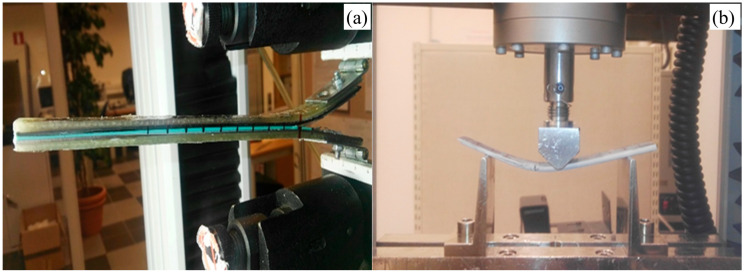
(**a**) DCB and (**b**) ENF test setup.

The test was carried out in displacement-controlled mode using Instron_3366 (USA, Norwood MA) with a 10 KN load cell for the DCB test and Instron_4411 (USA, Norwood MA) for ENF. The edge of the sample was marked with lines with a distance between them equal to 10 mm as a gauge for the crack extension (see Figure 5). The load was applied via hinges, and the test was interrupted after achieving an approximately 10 mm extension of the crack. Then the sample was unloaded with a constant rate of 5 mm/min and reloaded again with a constant rate of 2 mm/min according to [25]. The actual position of the crack tip in each step was marked on both edges of the specimen and was measured afterward with a precision-dial caliper, and then the average of those values was calculated. The loading with crack extension and the unloading were repeated eight times in order to get eight values for the loading and unloading curve for each specimen. The test was repeated on five specimens for each configuration. The standard outlines various data reduction techniques, including modified beam theory, compliance calibration method, and modified compliance calibration method. In this study, the compliance calibration (CC) method was selected for Mode II characterization. For the Mode I fracture toughness calculation (G_IC_), Equation (2) was used as presented in [24]. As explained in the standard, if the ratio of the opening displacement at delamination onset (δ) to the delamination length (a) exceeds 0.4, it is advisable to incorporate the large displacement correction factor (F) into the calculations (see Equation (3)).

To calculate the Mode II fracture toughness, we used Equation (5) as presented in the ASTM [26].

(2) $$G_{IC}=F\frac{nP_{max}\delta }{2ba}$$

(3) $$F=\left\{\begin{array}{c} 1,\;for\;\frac{\delta }{a}<0.4\\ 1-\frac{3}{10}{\left(\frac{\delta }{a}\right)}^{2}-\frac{3}{2}\left(\frac{\delta t}{a^{2}}\right),\;for\;\frac{\delta }{a}\geq 0.4 \end{array}\right.,$$

(4) $$n=\frac{\log(\frac{\delta_{i}}{P_{i}})}{\log a_{i}}$$

(5) $$G_{IIC}=\frac{3m{P_{max}}^{2}{a_{o}}^{2}}{2b}\;$$

In Equations (2) and (5), *b* is the width of the DCB specimen, ($P_{max}$) is the maximum applied load during one step of extension, t is the distance that can be calculated from the center of the piano hinge to the one-quarter of the thickness of the sample, m is the slope obtained from the regression analysis, and n is the compliance calibration factor (as can be extracted from the least-squares plot of the logarithm of the compliance ($\delta_{i}/P_{i}$) versus the logarithm of its respective crack length $a_{i}$).

## 4. Results and Discussion

The fracture toughness of Mode I and Mode II was determined through DCB and ENF tests, respectively. This section compares various parameters, including types of adhesives (ET445 FAST resin and Sika Power-533), adhesive thickness, types of steel surface (the notation for coated is cS and for uncoated uS) and UD composite (notation C), and adherend configurations (C–C) for composite–composite and C–cS or C–uS for the hybrids. The used notation for resin types is “ET” (prepreg) and “Sika”.

### 4.1. The Effect of Adhesive Type and Thickness on G_IC_

**Figure 6:**
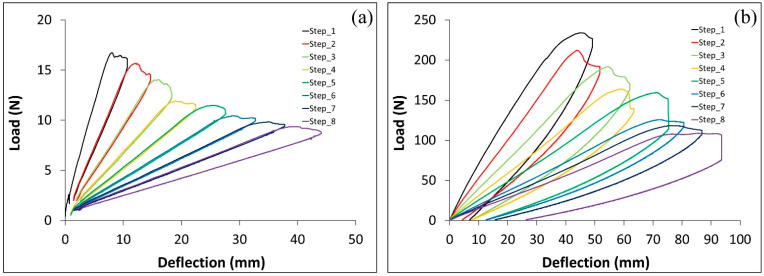
Load deflection curves in DCB test of a C–cS joint with (**a**) ET adhesive and (**b**) Sika adhesive.

**Figure 7:**
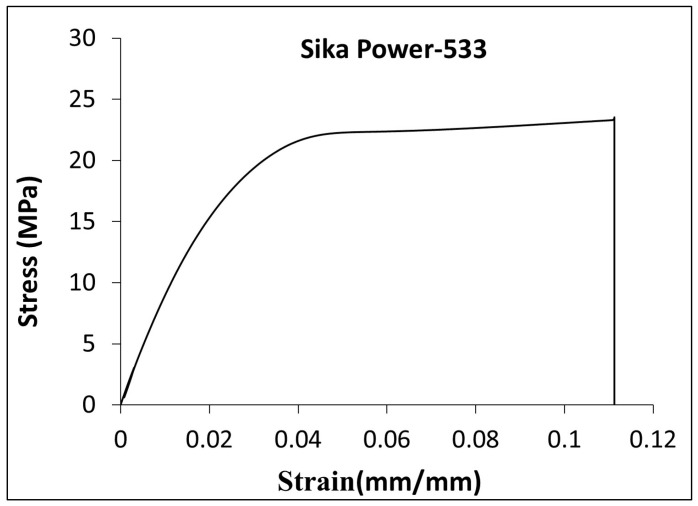
Stress–strain curve for Sika Power-533.

The effect of the adhesive type on G_IC_ from the DCB test is presented in this section. Typical load vs. deflection curves from the DCB test for specimens with ET and Sika adhesives are presented in Figure 6. The tests were conducted on C–cS DCB specimens. As illustrated in Figure 6, the force needed to propagate the crack was reduced with increasing the crack length. The force required to propagate the crack in the Sika adhesive was by an order of magnitude higher than that in the ET adhesive. It can be noted that the load deflection curve of DCB specimens with the Sika adhesive shows some irreversible deformation as well as overlapping between the loading/unloading curves, as shown in Figure 6b. This can be attributed to plastic deformation induced in the adhesive (hence residual deformation) and partial recovery (overlapping) occurring in the adhesive during the unloading step. Such performance is common for materials exhibiting non-linear behavior (e.g., time-dependent, viscoelastic, or viscoplastic). This is also evident from the stress–strain curve of Sika obtained from the tensile test of neat adhesive (as shown in Figure 7), the curve is highly non-linear with a well-defined yielding point. Although the tensile stress–strain curve for neat ET445 FAST resin is not available at this point, it is reasonable to assume that the matrix in such prepreg is more brittle with rather linear elastic behavior. Thus, there is another significant factor (mechanical performance/properties of adhesive) that is likely to have a strong influence on the results, probably much more important than the thickness of the adhesive layer.

**Figure 8:**
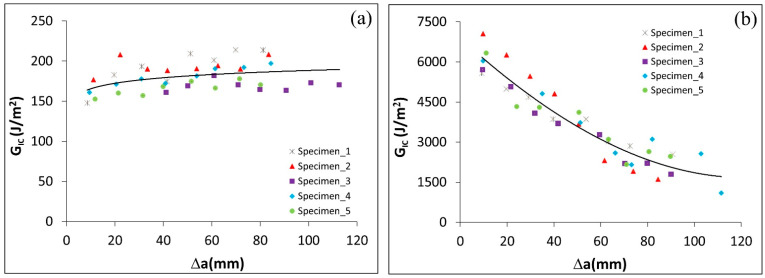
Critical energy release rate vs. $\left(∆a=a-a_{0}\right)$ for a hybrid joint C–cS, with (**a**) ET adhesive and (**b**) Sika adhesive.

The critical energy release rate in the ET adhesive layer case increased slightly with an increase of the crack length, as shown in Figure 8a. In the case of the Sika adhesive layer, the G_IC_ steadily reduced at each step, reaching a very significant change at the end of the test (the released energy at the 8th step is only 1/4 of the energy at the 1st step), when the crack length is increased (see Figure 8b). It can be interpreted that G_IC_ (as a material property) was measured for the DCB with prepreg resin as the adhesive, whereas for Sika, the constant value of G_IC_ was not achieved since it changed with crack length. A problem is also that the Sika resin is highly inelastic but we are using linear elastic stress analysis and a methodology based on LEFM. The difference between the performance of specimens with ET and Sika adhesives is very obvious from the load deflection curves, since the load level in the Sika specimen test was around 10 times higher than for ET samples. Similarly, the deflection (e.g., crack opening) was also higher for Sika specimens (around double that of ET specimens). This resulted in an extreme difference between the released energy in the ET and Sika adhesive layer cases: the C–cS joint with the Sika needed around 25 times the energy for the first crack propagation, and this difference was reduced to reach around 10 times for the last crack propagation step, However, these results should be interpreted with caution because not only the adhesive material was different in both cases but also its thickness. It has been shown in a previous study [28] that increasing the adhesive layer thickness six times leads to five times higher joint strength. Still, it cannot explain a 25-fold increase in energy for the crack propagation observed for Sika resin. Thus, it may be concluded that the properties of the adhesive are likely to affect the performance of DCB specimen more significantly than the adhesive layer thickness alone. On the other hand, the coated layer on the steel needs to be examined to determine whether it affects the joint strength. This will be addressed in the following section.

### 4.2. Effect of Coating on G_IC_ of DCB Specimens with Sika Adhesive

**Figure 9:**
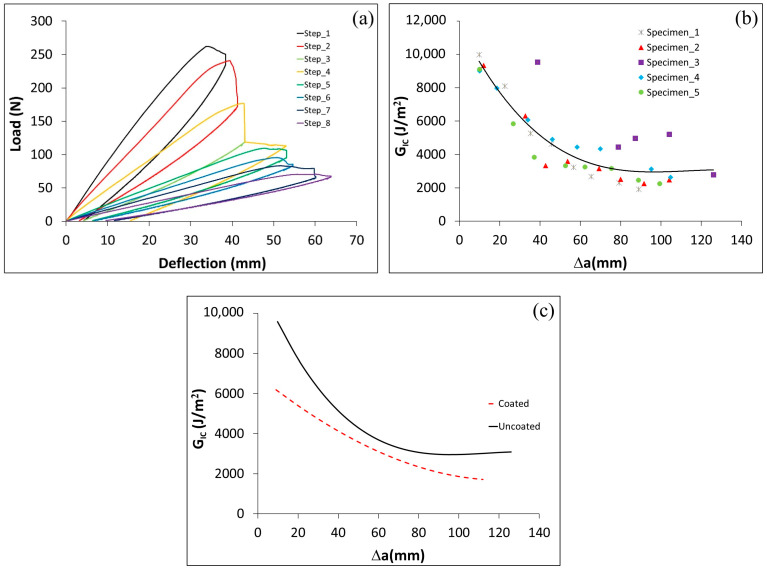
A hybrid C–uS joint with Sika adhesive. (**a**) Load deflection curve in DCB test, (**b**) $G_{Ic}$ vs. $\left(∆a=a-a_{0}\right)$, and (**c**) the critical energy release rate for coated and uncoated steel specimens.

To study the influence of the Al–Si coating on the performance of the joint, the load deflection of uncoated steel (Figure 9a) is compared with the coated steel presented in Figure 6b. The $G_{Ic}$ for uncoated steel was approximately 25% higher compared to coated steel (Figure 9b shows values for uncoated steel, Figure 9c the comparison between coated and uncoated steel). This could be attributed to the fact that the coating layer represents a weak region when dealing with Sika adhesive applications. Since uncoated steel performs well with a thick adhesive but not with a thin adhesive, it is necessary to evaluate the effect of the adherend surface type on joint strength when using a thin adhesive (ET). This will be examined in the next section.

### 4.3. G_IC_ of Joints with C-C and C-cS Adherends with an ET Adhesive Layer

**Figure 10:**
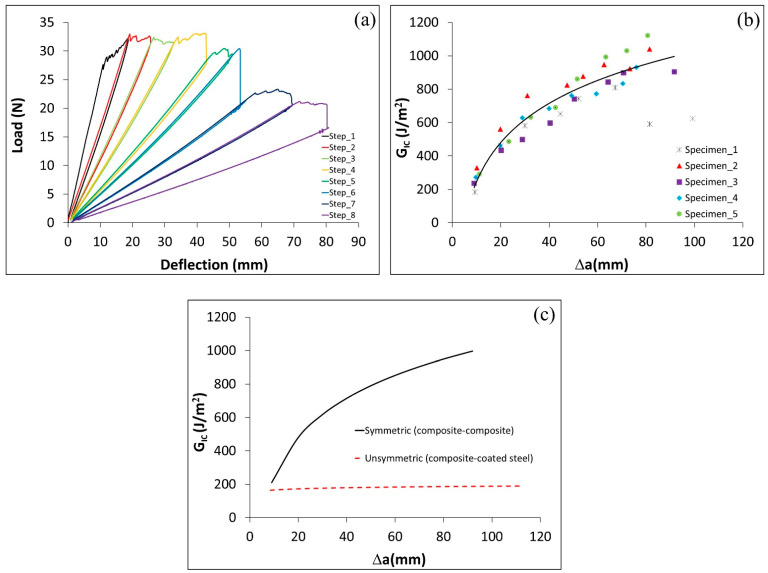
A C–C joint with ET adhesive. (**a**) Load deflection curve for DCB test, (**b**) critical energy release rate vs $\left(∆a=a-a_{0}\right)$, and (**c**) G_IC_ for C–C and C–cS adherends of DCB specimens.

This section examines the differences between symmetric C–C DCB specimens and hybrid specimens C–cS with an ET adhesive layer. The load deflection curves demonstrate that the C–C specimens required almost twice the load to propagate crack at the same crack length compared to C–cS specimens, as can be seen from the comparison of Figure 6a and Figure 10a. Furthermore, there was a significant improvement in the $G_{Ic}$ of C–C specimens over C–cS. In fact, the $G_{Ic}$ for C–C specimens was shown to be six times higher than that for C–cS specimens (see Figure 10b,c). The possible mechanisms for these differences are further analyzed in Section 4.5 using fractography.

### 4.4. G_IIc_ of ENF Specimens with C–C and C–cS Adherends and ET Adhesive

**Figure 11:**
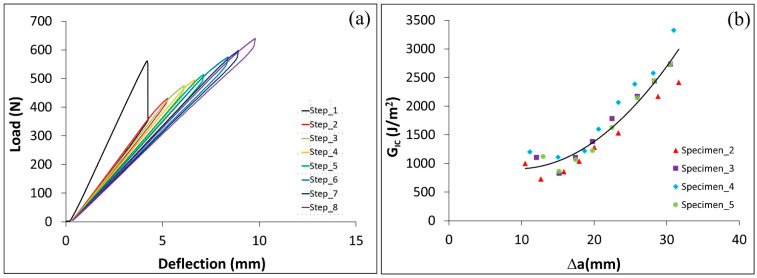
A C–C joint with ET adhesive layer. (**a**) Load deflection curve for ENF test and (**b**) $G_{IIc}$ vs. delta $\left(∆a=a-a_{0}\right)$.

**Figure 12:**
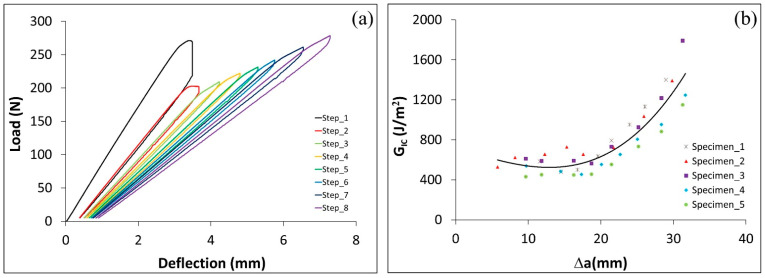
A hybrid C–cS joint with ET adhesive. (**a**) Load deflection curve for ENF test and (**b**) $G_{IIc}$ vs. $\left(∆a=a-a_{0}\right)$.

In this section, the effect of the type of adherends used in ENF specimens with ET adhesive on the energy necessary to propagate a crack ($G_{IIc})$ is discussed. Figure 11a and Figure 12a display the load deflection curves for C–C and C–cS, respectively. The results show that the load required to propagate the crack for (C–C) is around two times what is needed for (C–cS). Also, the $G_{IIc}$ for the C–cS specimens is around half of the C–C specimens as shown in Figure 11b and Figure 12b. As expected, the Mode II fracture toughness is significantly higher than for Mode I.

### 4.5. Fracture Surface and Failure Mode in the Joint (Fractography)

The fracture surfaces for all specimen types (DCB, ENF) were analyzed through visual inspections, with optical microscopy photographs provided in this section.

**Figure 13:**
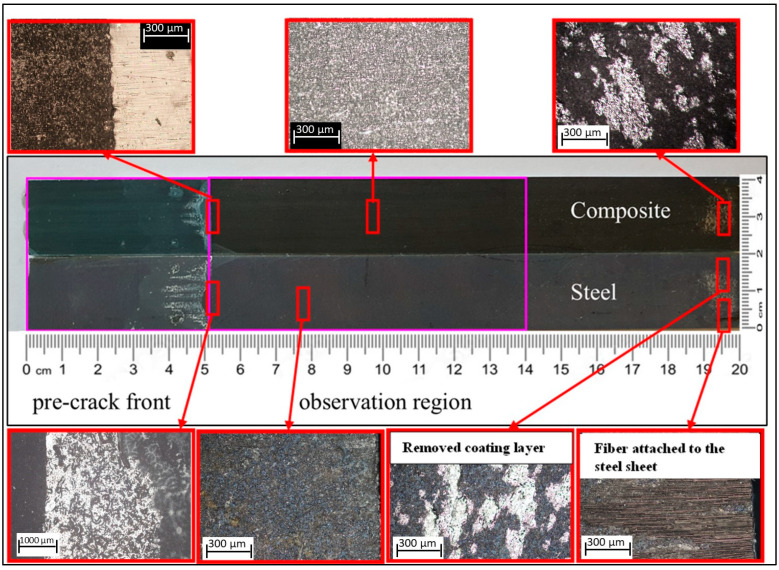
Fracture surface of a hybrid C–cS DCB specimen with ET adhesive layer after the test.

**Figure 14:**
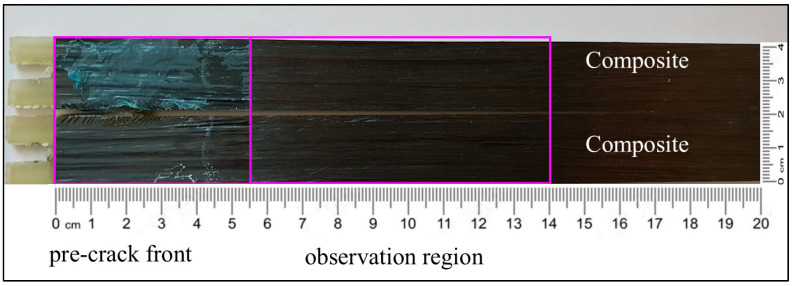
Fracture surface of a C–C DCB specimen with ET adhesive layer after the test.

**Figure 15:**
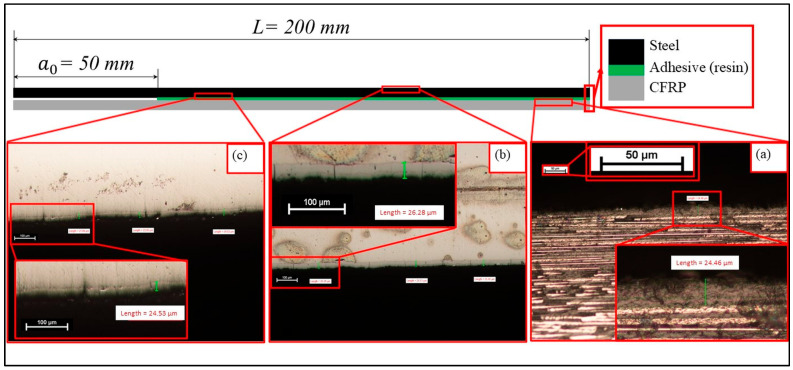
Sketch of a hybrid C–cS DCB specimen with an ET adhesive layer, indicating specific points for the microscopic image: (**a**) the surface of the composite part, which is in contact with the adhesive layer, contains the remaining ET resin; (**b**) the outer surface of the steel sheet; and (**c**) the inner surface of the steel sheet, which is in contact with the adhesive layer, shows only the coating layer on top of the steel, with no resin bonded to it.

In case of C–C and C–cS DCB samples with a thin ET adhesive layer, the results indicate that all the specimens exhibited failure according to the adhesive failure mode and the fiber-tear mode, as demonstrated in Figure 13 and Figure 14. As illustrated in Figure 13, the adhesive layer was completely separated from the steel sheet, which is evident from Figure 15b,c, where only the coating layer remains on the steel, while the resin layer was attached to the composite part, as depicted in Figure 15a. Additionally, a portion of the coated layer at the end of the sample was also separated from the steel sheet (manually breaking the specimen after the test), while in the case of composite adherends (C–C), some fibers were transferred between the two adherends due to fiber bridging, as shown in Figure 14. Fiber bridging impacts the mechanical behavior and energy dissipation in the material, as these fibers provide additional resistance to crack growth. This phenomenon can increase the fracture toughness (G_IC_) of the material, as more energy is required to extend the crack in the presence of bridging fibers. This is one of the reasons supporting the findings in Section 4.3, which indicate that G_IC_ for C–C specimens was approximately six times higher than that for C–cS specimens. This type of joint (C–cS) represents the weakest joint compared to the other configurations examined in this investigation. The presented results show that the interface of the coated steel/ET resin was the weakest link governing the G_IC_ values. This effect was even stronger in the uncoated steel case.

**Figure 16:**
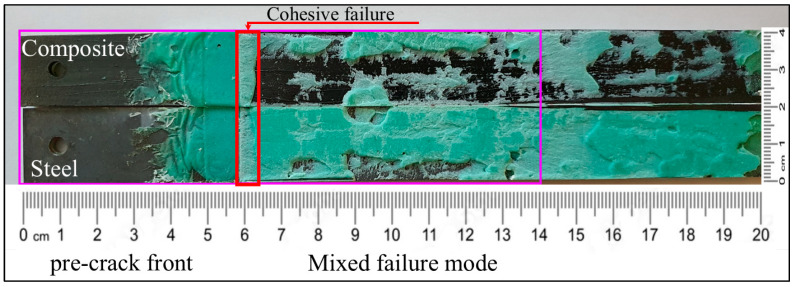
Fracture surface of the hybrid C–cS DCB specimen with thick Sika adhesive.

In case of C–cS DCB bonded by the Sika adhesive, three different types of failure were observed: cohesive failure, adhesive failure, and mixed failure modes (see Figure 16). The cohesive failure mode (with adhesive present on both substrates after the separation of that area) manifested at the very beginning of the crack propagation, due to placing a non-adhesive insert in the middle between the substrates in order to create an initial pre-crack in the middle of the adhesive. This type of failure was present for a few millimeters, and then the behavior of the failure changed to a mixed failure mode (adhesive failure and cohesive failure mode) that covered the rest of the failure area, as shown in Figure 16, in which most of the adhesive failures occur at the composite surface.

**Figure 17:**
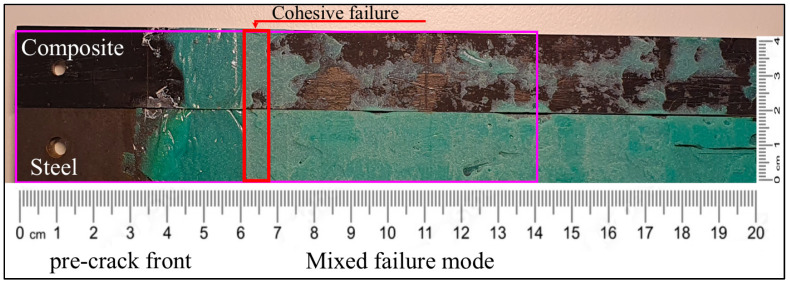
Fracture surface of the hybrid C–uS DCB specimen with Sika adhesive.

Similar behavior of the failure mode was present in the uncoated steel strip with Sika adhesive because the crack mainly propagated at the adhesive/CFRP interface (see Figure 17). This means that in both cases the Sika/composite interface was the weakest link for failure and whether the steel was coated or not had a minor influence. That confirms the conclusion in Section 4.3 that the fracture toughness of the joint was almost independent of the presence of the coating.

**Figure 18:**
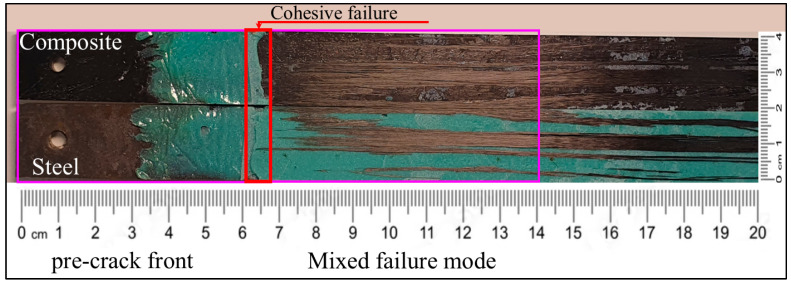
Fracture surface of the hybrid C–uS DCB specimen with Sika adhesive showing fibers bridging the crack.

In one of the C–uS specimens, the composite adherend exhibited a delamination failure mode (see Figure 18). The crack propagated deeply into the composite adherend and fiber bundles were present on the steel part. This indicates that the fracture toughness of the S/Sika interface was larger than that of the composite.

**Figure 19:**
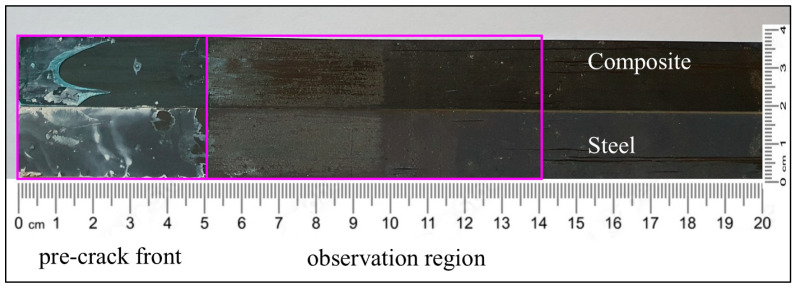
Fracture surface of a hybrid C–cS ENF specimen with ET adhesive.

For C–cS ENF specimens with ET adhesive, the failure occurred within the coating layer of the steel adherend, not at the interface between adherends. The failure was similar to a cohesive failure but within the coating layer instead of the adhesive layer, as shown in Figure 19.

## 5. Conclusions

In the presented paper, two methods of joining steel with unidirectional composite have been compared. In the first case, a tough adhesive with non-linear behavior, Sika Power-533, formed a thick adhesive layer when already produced steel and composite parts were joined. In the second case, the excess resin (ET445) from the composite prepreg, presumably showing liner elastic, brittle behavior, was utilized as the adhesive, and the curing of the composite and the joining of adherends were performed in one step. The load deflection curves and values of critical energy release rate $G_{c}$ for DCB and ENF tests were analyzed. This was carried out for composite–composite (C–C) and composite–steel (C–S) specimens with coated and uncoated steels which were made using two different polymers as adhesives. The following conclusions were drawn from these results:

The properties of the adhesive considerably affected the force required to propagate a crack in DCB tests and had a significant effect on measured values of the $G_{Ic}$. In specimens with the Sika Power-533 adhesive that is designed to be compatible with the steel and the composite, it required ~25 times more energy to start crack propagation compared to ET445 specimens. However, at later stages of crack propagation, this energy ratio decreased to around 10 times (at the final crack propagation step).

The critical energy release rate for DCB specimens with the thin ET adhesive increased slightly as the crack length increased, whereas for Sika adhesives, G_IC_ steadily decreased at each step, showing a significant change by the end of the test. This suggests that the crack propagation mechanisms changed as the crack length increased, likely due to the inelastic behavior of the polymer. This may be attributed to the yielding of the Sika and formation of the plastic zone at the crack tip, which assisted crack propagation at the longer crack length. This finding is consistent with the results reported in [29].

Both coated and uncoated steel with the Sika adhesive showed similar load deflection trends, while $G_{Ic}$ for the uncoated case was about 25% higher than for the coated case. The coating layer acted as a weak region in thick adhesive applications, reducing the joint’s ability to resist crack propagation.

The symmetric (composite–composite) specimens exhibited much stronger performance, both in terms of load-bearing capacity and energy release rate, compared to composite-coated steel specimens with ET445 adhesive.

Fractography showed that the Sika/composite interface was the weakest link in a C–S joint, with almost zero effect of the coating. Cohesive failure was observed in the early stage of the crack propagation but shifted to a mixed failure mode (adhesive and cohesive) as the crack progressed. As expected, using the ET445 adhesive the “weakest link” was the surface of the steel since almost no residuals of the ET445 were found on the steel part of the fracture surfaces.

## Figures and Tables

**Table 1:** Steel, CFRP, and Sika Power mechanical properties.

Material Type	Mechanical Properties	Value
steel	Young’s modulus	206 GPa
	Poissons’s ratio	0.3
UD T700/E445 CFRP	Longitudinal tensile Young’s modulus	113.6 GPa
	Longitudinal tensile strength	1901 MPa
	Transverse tensile Young’s modulus	7.7 GPa
	Transverse tensile strength	27.6 MPa
	Longitudinal compression Young’s modulus	111.7 GPa
	Longitudinal compression strength	923.2 MPa
	Transverse compression Young’s modulus	8.7 GPa
	Transverse compression strength	140.5 MPa
	Curing conditions	7 min at 160 °C & 3 bar
Sika Power-533 MBX	Young’s modulus	∼850 MPa
	Tensile strength	20 MPa approx.
	Elongation at break	~20%
	Glass transition temperature	~95 °C
	Curing conditions	20 min at 175 °C

## Data Availability

Data will be available when it is requested.
